# Perceptions of palliative care in a South Asian community: findings from an observational study

**DOI:** 10.1186/s12904-020-00646-6

**Published:** 2020-09-14

**Authors:** Naheed Dosani, Ravi Bhargava, Amit Arya, Celeste Pang, Pavinder Tut, Achal Sharma, Martin Chasen

**Affiliations:** 1 Division of Supportive and Palliative Care, Brampton Civic Hospital, William Osler Health System, 2100 Bovaird Drive East, Brampton, Ontario Canada; 2 Division of Palliative Care, Faculty of Health Sciences, McMaster University, Hamilton, ON Canada; 3 Corporate Department of Research, William Osler Health System, Brampton, ON Canada; 4 The Global Institute of Psychosocial, Palliative and End-of-Life Care, University of Toronto, Toronto, ON Canada

**Keywords:** Palliative care, Cultural competence, Social determinants of health, Palliative perceptions, South Asian community

## Abstract

**Background:**

Patients often view “palliative care” (PC) as an approach that is synonymous with end-of-life and death, leading to shock and fear. Differing cultural and social norms and religious affiliations greatly determine perception of PC among diverse populations.

**Methods:**

This prospective observational study aimed to explore perceptions of PC among South Asian community members at one Canadian site. Patients who identified themselves as being of South Asian origin were consented and enrolled at a PC Clinic at a community hospital in Brampton, Ontario serving a large South Asian population. Participants filled out an 18-question survey created for the study and responded to a semi-structured interview consisting of 8 questions that further probed their perceptions of PC. Survey responses and semi-structured interviews content were analyzed by four authors who reached consensus on key exploratory findings.

**Results:**

Thirty-four participants of South Asian origin were recruited (61.8% males), and they were distributed by their age group as follows: [(30–49) - 18%; (50–64) – 21%; (65–79) - 41%; (≥ 80) – 21%]. Five main exploratory findings emerged: (i) differing attitudes towards talking about death; (ii) the key role of family in providing care; (iii) a significant lack of prior knowledge of PC; (iv) a common emphasis on the importance of alleviating suffering and pain to maintain comfort; and (v) that cultural values, faith, or spiritual belief do not pose a necessary challenge to acceptance of PC services.

**Conclusions:**

Observations from this study provide a source of reference to understand the key findings and variability in perceptions of palliative care in South Asian communities. Culturally competent interventions based on trends observed in this study could assist Palliative Physicians in delivering personalized care to South Asian populations.

## Background

The integration of palliative care for individuals with serious, life-limiting illnesses has the potential to improve quality of life for patients and families, particularly when introduced early on in the illness trajectory [1], while reducing healthcare costs [2] and in some cases increasing life expectancy [3]. Yet, despite its many advantages, unfortunately several barriers still exist to accessing these services.

One highly prevalent barrier is the public perception of palliative care (PC), and even more specifically, “persisting definitional issues” which cause delays in the initiation of a PC approach, and in some cases, referrals to PC services [4]. This definitional issue may be at least partially attributed to the initial perceptions that individuals hold prior to receiving care. Commonly, patient populations view “palliative care” as an approach that is synonymous to end-of-life and/or care close to death, and thus PC is often met with shock and fear when introduced [5]. Yet, studies have shown that patients and caregivers who receive palliative care interventions early in the trajectory of their illness, usually feel “more comfortable”, showcase a broadened understanding towards PC, enjoy an improvement in quality of life, and feel more equipped with resources to cope [5].

As public attitudes towards end-of-life care and PC are complex [6], it is thought that cultural characteristics including social structures and religious affiliation [7–11] must be addressed to improve public awareness. It has been reported that people are divided about the role of palliative care due to faith-based beliefs depending on how they view death [11]. Some believe in full autonomy when it comes to life and death situations, and others view palliative care as replacing religious authority, resulting in a contrasting dichotomy. These beliefs, combined with a poor understanding of palliative care, lead to low enrolment into palliative care services within certain communities [12]. Despite this previous work, there is a paucity of research that has measured cultural perceptions of PC among members of South Asian communities. Some studies have shown that immigrants of South Asian origin have the highest risk of aggressive end-of-life care, when compared to immigrants from other regions of the world [13].

Our observational study was designed to learn more about the perceptions of PC in an area of Ontario, Canada, where the per capita population of South Asian immigrants and peoples of descent is over 30% [14]. Therefore, insight into the perceptions of PC within this population, and the identification of barriers to accessing care were thought to be necessary to identify those individuals who would benefit from a PC approach. This insight has the potential for the development and implementation of culturally competent PC services and understanding among healthcare professionals.

## Methods

Data was obtained over a 3-month period from surveys and semi-structured interviews with self-identified South Asian patients receiving clinic-based, outpatient specialty PC at William Osler Health System in Brampton, Ontario, Canada. Institutional ethics approval was granted by the Research Ethics Board, and informed consent was obtained from all study participants. The study population included adult patients newly referred to out-patient PC services (clinic or home visits). Inclusion criteria were: (i) self-identified members of a South Asian community; (ii) able to communicate in English, Punjabi, Gujarati or Urdu. Patients were excluded from the study if (i) under the age of eighteen; (ii) had no life-limiting diagnosis; and (iii) were not eligible for PC. This research was approved by the Department of Family Medicine, McMaster University and was supported by their annual pilot research grant funding.

Study participants, and their family members and/or caregivers were approached prior to their first PC consultation. The participants seen in clinic were approached in person, while patients having appointments at home were contacted by phone. The study team was introduced to the patients by the clinic nursing staff prior to their appointment. If the patients agreed to be seen by the interviewers, the study team then presented the details of the study to them. Patients were informed that their responses would be anonymized and would not have any impact on the care they receive from their physician. After explaining the study and obtaining informed consent from individual patients, research assistants administered a survey and semi-structured interview (Additional file 1). A thorough literature search was conducted on perceptions of palliative care [4–12], and survey and interview questions design were informed by this literature. The survey was an expedient way to gather basic demographic data and establish a baseline of participants’ perceptions of palliative care, while interviews allowed the authors to probe further and for participants to reflect and share their thoughts in their own words. The aim of the twenty-question survey was to gain demographic information and gauge overall knowledge about participant perceptions of PC. The survey was divided into three domains: (i) demographics; (ii) knowledge of PC, and (iii) individual preferences regarding PC. Surveys conducted in the clinic were completed by participants themselves, while surveys for patients having appointments at home were administered by the research assistants over the phone. The administration of the survey took approximately 10 min.

Following the survey, participants were administered a semi-structured interview. Eight guiding questions were developed to probe more deeply into participants’ perceptions of PC. The interview was divided into two components. In the first component, existing knowledge of PC and sources of this knowledge were explored. A second component probed perceptions of PC and ideals surrounding care, including perceptions related to existing social support systems and living situations, and thoughts on end-of-life care and a “good death”. Interview questions included: “Can you please describe to me what you know about palliative care”; “Where and when did you learn about palliative care”; “Who is caring for you right now? Who would you like to be caring for you at this time?”; and “In an ideal world, when would you first discuss end of life care option with your doctor”. The interviews lasted between 10 and 20 min per patient. While considerably shorter than traditional semi-structured interviews, this length reflected the time each participant had available, as well as participant reticence in expanding upon interview topics regarding sensitive health and care concerns and end of life themes. Our team did not wish to burden participants, and given the exploratory nature of the study interviews nonetheless provided valuable additional insight.

Surveys were designed in English and translated into Punjabi and Urdu. Interviews were conducted in five languages: English, Punjabi, Urdu, Hindi, and Gujarati, as best suited to the participant. Where participants consented, interviews were also audio recorded. All interviews were subsequently transcribed, with non-English interviews translated by a certified translator.

### Data analysis

Survey responses were tabulated, and qualitative data collected through semi-structured interviews for all patients was analyzed by authors CP, ND, PT and AA. These four team members—the research coordinator, research assistant, and palliative care physicians—brought diverse backgrounds to their interpretation of the data. Inductive thematic analysis [15, 16]; was used to analyze the data. Each author read and reviewed interview transcripts for key themes. Results were then collated and discussed by the authors, until consensus was achieved. Collated results were then communicated and reviewed by the group, and a consensus was achieved on the final themes emergent in the interviews. Together with the survey results, five main exploratory findings were identified.

## Results

### Participant demographics

A total of 34 participant groups were recruited for this study. While the study initially sought to examine the perceptions of individual patients via isolated interviews, during implementation, we found that all of our patients preferred to have their family members accompany them due to the sensitive nature of the content. While survey data reflects individual patients recruited, we thus find it more appropriate to conceptualize a “participant group” in relation to the qualitative data. In all cases, interview questions elicited responses from individual patients, who participated as the sole participants or answered in concert or dialogue with family members.

**Fig. 1:**
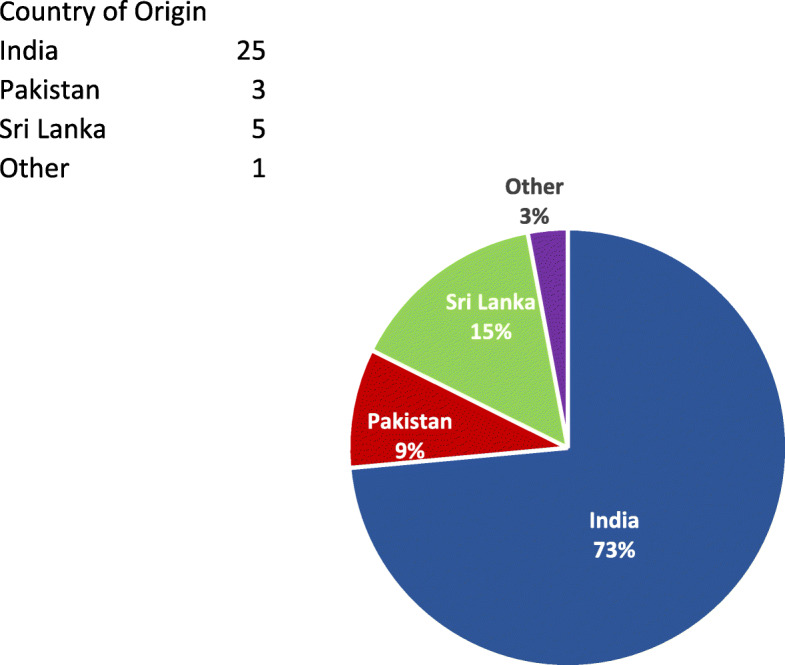
Country of Origin. Legend: India (*n* = 25), Pakistan (*n* = 3), Sri Lanka (*n* = 5), Other (*n* = 1)

**Fig. 2:**
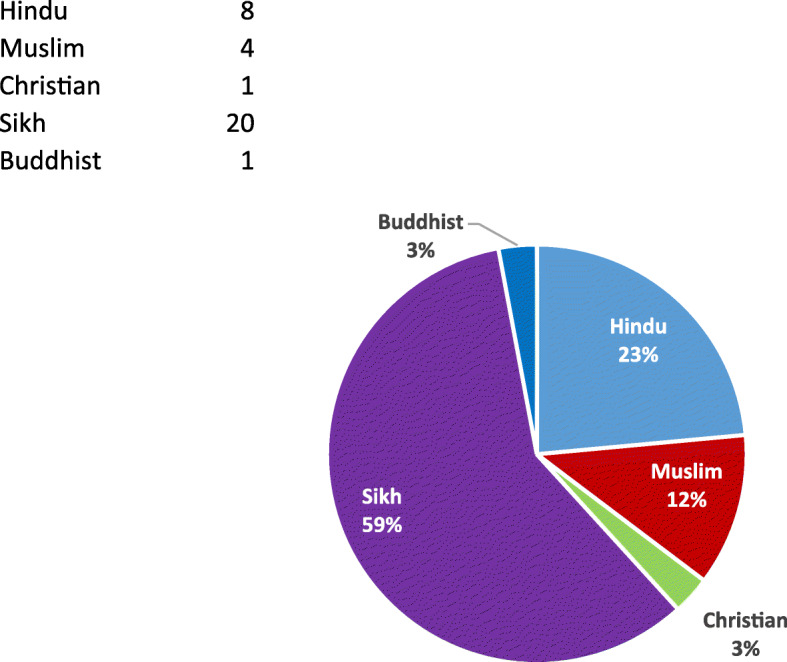
Religious Affiliation. Legend: Hindu (*n* = 8), Muslim (*n* = 4), Christian (*n* = 1), Sikh (*n* = 20), Buddhist (*n* = 1)

As Table 1 shows, individual patients included both men and women, with an age range from thirty to eighty years and above. The majority were of Indian origin (Fig. 1) and identified their religious affiliation as Sikh (Fig. 2). Some participants chose not to respond to select survey questions. Of note, all patients had a diagnosis of cancer.

**Table 1:** Demographics of Participants

**Total number of participants (*n*)**	34
Sex (*n*)	
Male (%)	21 (61.8%)
Female (%)	13 (38.2%)
Age range (years)	
30–49	6 (17.6%)
50–64	7 (20.6%)
65–79	14 (41.2%)
≥ 80	7 (20.6%)
Religious Identification	
Hindu	8 (23.5%)
Muslim	4 (11.8%)
Christian	1 (2.9%)
Sikh	20 (58.8%)
Buddhist	1 (2.9%)
Country of Origin	
India	25 (73.5%)
Pakistan	3 (8.8%)
Sri Lanka	5 (4.7%)
Other	1 (2.9%)
Preferred Language of Communication	
English	11 (32.4%)
Punjabi	13 (38.2%)
Urdu	4 (11.8%)
Tamil	2 (5.9%)
Invalidated^a^	4 (11.8%)
Highest level of education	
Grade school	3 (8.8%)
Some high school	5 (14.7%)
High school graduate	4 (11.8%)
Some College/University	2 (5.9%)
Trade/Technical/vocational training	1 (2.9%)
College/University Graduate	9 (26.5%)
Post Graduate Degree	4 (11.8%)
No Education	5 (14.7%)
Invalidated^a^	1 (2.9%)
Years since immigrated to Canada	
0–5 years	6 (17.6%)
6–10 years	5 (14.7%)
11–15 years	3 (8.8%)
16-20 years	6 (17.6%)
20+ years	14 (41.2%)

^a^ Invalidated – no response, or multiple selections nullifying response

### Emergent findings

Five main findings emerged from the data: (i) differing attitudes towards talking about death; (ii) the key role of family in providing care; (iii) a significant lack of prior knowledge of PC; (iv) a common emphasis on the importance of alleviating suffering and pain to maintain comfort; and (v) that cultural values, faith, or spiritual belief do not pose a necessary challenge to acceptance of PC services.

### Differing attitudes towards death

Among respondents there was a range of responses to the question of what a good death means to them. Many participants expressed nervousness around questions related to death, and all responses were brief. In two cases, family member participants had adverse reactions, gasping and becoming concerned about the state of their family members’ health.

Among others, there was frank acceptance. For instance, one middle-aged patient participant expressed: “Well just as long as you’re peaceful and happy with your family, you know. When you got to go, you gotta go.”

### The key role of family in providing care

The importance of family members providing care was a central discussion point for many participants. Participants described family support systems that are often made up of multiple members, including spouses, adult children and their spouses, and grandchildren. Responsibility for care was often described as being shared amongst family members. Many respondents were currently living with family members, many in multi-generational households. Further, most respondents were accompanied by family members to their PC appointments.

When asked who is caring for them right now, and who they expect to be caring for them in the future, the vast majority of participants named their family members and expressed a desire to continue to receive support from their family, ideally in their home.

### A significant lack of prior knowledge of PC

There was a significant lack of understanding and prior knowledge of PC among study participants. At the outset, only 70 % of participants had ever heard of PC. Meanwhile, of those who had heard of PC: 80 % were slightly knowledgeable, 10 % were moderately knowledgeable, and 10 % were very knowledgeable.

In response to the interview question “could you please describe to me what you know about palliative care” responses included: “I have no idea”, and “No, I don’t know. It could be a treatment, but I don’t know what kind of treatment it is.” One participant responded:

“I have no idea before. And when my mom got sick… but I haven’t, no idea. Only I know that people they’re getting older, and then no one take care of them, and then they send them to a nursing home, and then…that’s the end of their life. But uh, I never know anything.”

Respondents also conveyed perceptions of palliative care that were incongruent with current standard definitions. For instance, one patient participant stated: “I don’t really know much about it. Because I heard that...it’s something that there’s no more treatment, and then you go palliative care...”.

Among caregivers who responded with individual patients, there was a range of knowledge of PC, nonetheless it was still very limited overall. Where caregivers had heard of PC, it was through past experiences with family members, through friends, or some familiarity because of their field of work. Almost three quarters of participants believed that more information about PC should be generally available.

### An emphasis on alleviating suffering and maintaining comfort

Participants emphasized that they wanted to avoid or alleviate suffering, which was consistent when discussing the entire trajectory, including end-of-life. In response to the question regarding a good death, one participant stated: “With family, without any pain”; while another stated that ideally there would be no suffering. Reflecting this sentiment, one son responded:

“…we hope she is not suffering so much…that it would not hurt so much, because this medication can help her relief a little bit.”

Indeed, when participants were asked to indicate what best matched their understanding of the term “palliative care”, the most common perception of the term was as “comfort measures only”, followed by “total care of a patient with a life limiting illness”. Further, participants held common perceptions of benefits of PC, and few perceptions of risk. Participants by and large associated the benefits as “better quality of life” and “less suffering”. More than half of participants also associated PC with “more resources provided for patient and family”. None associated it with hastened death, as seen in the responses to the survey (Table 2), and the majority of respondents did not have any associations of risk.

**Table 2:** Additional Survey Responses

Respondents on whether they have heard of Palliative Care	
Yes	10 (30.3%)
No	23 (69.7%)
If yes to above, level of knowledge^a^	
Slightly Knowledgeable	8 (80%)
Moderately Knowledgeable	1 (10%)
Very Knowledgeable	1 (10%)
Participant understanding of the term – Palliative Care	
Stopping medical treatment only	1
Comfort measures only	10
Total care of a patient with a life limiting illness	9
Symptom management of a life limiting illness	5
Medical assisted aid in dying	2
Other:	
Pain Management	2
End of Life	2
Invalidated^c^	1
Source of information regarding Palliative Care prior to receiving this survey ^b^	
Not applicable	17
Family Physician / Primary care provider discussed it	9
Close friend or relative’s experience with Palliative Care	4
Know of someone involved or received Palliative Care	2
Conversation with friend / family / acquaintance	0
Employment in health care	0
Media (Radio, Television, Newspaper, Magazine, Website, Social media)	0
Other	4
Perceived benefits associated with palliative care^b^	
Better quality of life	17
Less suffering	18
Longer life span	11
More resources provided for patient and family	15
Hastened death	0
Other (Don’t know/Unspecified)	5
Perceived risks associated with palliative care ^b^	
Worsened quality of life	3
More suffering	3
Shorter life span	4
Stopping treatment	1
Giving up hope	1
Other (Don’t know/Unspecified)	19
Invalidated^c^	6
Perceived setting to receive palliative care^b^	
Home	29
Hospice	2
Hospital	14
Nursing home / Long-Term Care residence	2
Other	0
Perceived medical professional responsible for providing palliative care^b^	
Nurse	21
Doctor	24
Family/Partners/Relatives	12
Homecare Providers/Caregivers/Personal Support Worker	7
Other	2
Perceived most responsible decision maker for Palliative Care Decisions	
Family Member	10
Family Doctor	4
Patients	7
Specialist	2
Multiple Responses	11
Time point in illness trajectory that participants consider ideal to receive information about Palliative Care	
I would not want information at any point	1
I would like more information to be generally available	25
I would only want information to be provided if diagnosed with a life threatening illness	3
I would only want information if a life threatening illness became a terminal diagnosis	4
Invalidated^c^	1
Discussion about death and dying in the community	
Not enough	23 (67.6%)
About the right amount	5 (14.7%)
Too much	6 (17.6%)
Whether Palliative Care aligns with cultural values and/or faith/spiritual beliefs	
Yes	9 (26.5%)
No	15 (44.1%)
Unsure	10 (29.4%)
Importance of religion or spiritual belief to participants in Palliative Care	
Not important	5 (14.7%)
Important	22 (64.7%)
Unsure	7 (20.6%)

^a^ Subset of Patients who answered yes to question “Have you heard of Palliative Care?”

^b^ Patients selected as many options as applicable to them, therefore total number of responses is greater than number of participants

^c^ Invalidated – no response, or multiple selections nullifying response

### Consistency with cultural values, faith, or spiritual beliefs

**Fig. 3:**
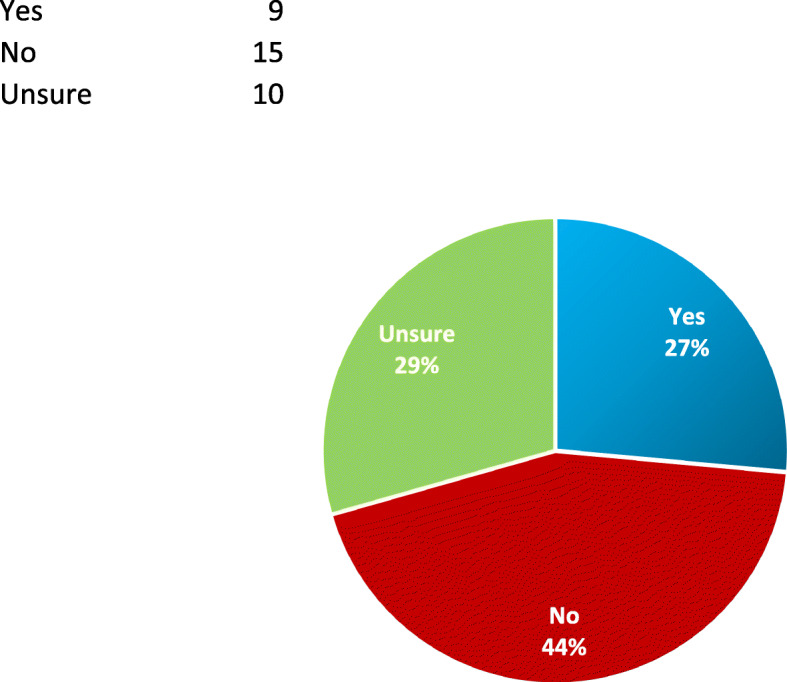
Is PC Consistent with your Cultural Values and/or Faith/Spiritual Beliefs? Legend: Yes (*n* = 9), No (*n* = 15), Unsure (*n* = 10)

Variable responses were observed on whether PC services were consistent with cultural values, faith, or spiritual beliefs among this South Asian community as seen in Table 1. Twenty-eight percent of participant groups agreed that PC is consistent with their values and beliefs, while 44 % understood PC to be against their values and beliefs. Twenty-nine percent of the participant groups were unsure (Fig. 3).

## Discussion

Recent research in Ontario, Canada, has demonstrated that immigrants originating from South Asia have the highest risk of aggressive end-of-life care, when compared to those born in other regions of the world [13]. Further to these findings, our study has observed a significant lack of knowledge and awareness regarding PC among South Asian study participants. The lack of knowledge in patients regarding PC and end-of-life care was shown to be prevalent in close to 50% or more of the studied population in multiple sources [17–19], something we observed in 70% of our South Asian patient population. The gap in knowledge regarding PC is likely to be at least a contributing factor resulting in the increased likelihood of aggressive end-of-life care in the South Asian population, a trend also noted in other racialized populations and immigrant communities [13, 20–22]. This disconcerting trend of lack of awareness regarding Palliative Care has remained alarmingly high for the past 15 years.

It was telling that many of our study participants wanted to avoid pain and suffering and associated PC with better quality of life and family supports. None of our participants explicitly voiced a desire for aggressive or futile interventions at end-of-life. All of these are treatment goals consistent with the key principles of palliative care, whose components include symptom management and monitoring, discussions about advance care planning and goals of care, and extra support for both the patient and family via an interdisciplinary team [1, 23]. Yet, once again, most of our study participants (70%) demonstrated a lack of knowledge about PC and 44% understood PC to be against their values and beliefs. The vast majority also expressed a desire for more information about PC. This incongruence may be part of a larger trend, which has been reported in other racialized communities and immigrant groups as well [20, 22]. Further investigation is needed into the causes of these differences, which may reveal why South Asians receive aggressive care at end-of-life that is often counter to their wishes.

Our findings further demonstrate the fact that the role of family in providing care near the end of life is key for this patient population, especially as many participants live with children and grandchildren in multi-generational households. This preference has also been observed in previous research in South Asians populations [24] and even other minority groups. For example, research in African American, Latin American, Chinese American, and African Canadian populations has shown that PC and end-of-life care may be better delivered via a family-centered approach [25–27]. This approach may differ from the autonomy focused approach that has been noted to be a preference of those born in Canada, as well as other Western countries [28]. In fact, various ethnic groups would be inclined to involve their family in decision making, although this would need further study in our specific patient population.

The great variability noted in patient attitudes and responses when discussing PC options and their understanding of the meaning of death and end-of-life itself, was telling. In effect, both ends of the spectrum, from denial to acceptance were noted in patients of South Asian origin. This variability in patient views PC and end-of-life care is consistent with findings among multiple minority populations [17–19, 26, 29–32]. Our patient population of South Asians is widely heterogenous, with diverse religious, cultural and linguistic preferences, which alongside idiosyncratic preferences, speaks to this variability in responses. The role of heterogenous cultural values, and spiritual beliefs in the South Asian population needs to be further examined with respect to preferences at end-of-life, particularly as previous research has specifically focused on disparities in communication.

### Clinical implications

As an observational study, our findings contribute to an understanding of perceptions of PC in a population of patients who are not extensively studied. The study’s results further indicate the great potential to improve and increase public knowledge and awareness of PC among this population. While participants had little background knowledge of PC, most participants expressed a willingness and desire to learn more about PC and even expressed preferences for treatment consistent with palliative approaches to care. Therefore, culturally competent interventions to assist healthcare professionals with the introduction of PC and difficult conversations are essential to establish rapport and improve patient understanding.

### Study limitations

Limitations of this study include the diagnosis of cancer in all patients in this study due to which, the findings of our research could vary in the context of other life-limiting illnesses of a non-malignant nature. We must note that this is a pilot project with a sample that cannot be generalized. Furthermore, uncertainties may have arisen when caregivers were present with study participants during the interview process, which may have made it more challenging to discern the individual perceptions of PC of patients alone. In addition, interviews conducted with respondents prior to their appointments with the PC team, may have been limited by the fact that participants unintentionally tailored responses due to possible associations between the research team and potential care providers. Patients who did not participate in the study may have done so due to doubts or concerns regarding PC. As a result, the responses may not still encompass the full range of perceptions that exist in the South Asian community. The amount of time the researchers had to conduct these interviews was also highly variable, and dependent on the patient’s availability. The interviews were limited to less than 20 min so as to not cause patient fatigue or burden them prior to their consultation with the PC Physician. This also directly limited the amount of subjective information that could be obtained from each patient. The study also did not account for certain specific demographics such as socio-economic status, and hence was unable to comment on attributing the variance in responses to the different demographic factors of all participants. Given the large immigrant population within this community, collecting information on the immigration status of patients could have also shed light on salient differences in between new-comers’ views to palliative care in comparison to second Generation Canadians. As has been reported, new immigrant patients are more likely to receive end-of-life care which is not in line with their wishes as compared to the non-immigrant populations [18]. Lastly, the survey conducted in this study was a cursory approach to identify the trends of perception of the South Asian population towards PC. Literature suggests that surveys are not well suited to gain insight into attitudes to complex and indeterminate issues [6].

Future research could consider collecting data distinctly from patients and caregivers, in order to systematically consider disparate perceptions. Broader dissection of the vocabulary used by South Asian community members (for example, words or phrases used to describe pain, serious illness or death) and how these impact clinical assessments could also be investigated. Finally, the evaluation of strategies to improve the introduction of PC as a topic for South Asian patients and the uniquely complex and challenging conversations associated with end-of-life could be a useful next step in this area of study.

## Conclusion

This study highlights important barriers that are present in accessing PC. Through further public outreach and education initiatives that are both culturally sensitive and delivered in a range of languages, there is great potential to increase awareness of palliative approaches to care. If this gap is bridged, it may lead to greater appreciation and uptake of PC services in the South Asian population. Mixed findings on cultural values, faith, and spiritual beliefs in the context of PC within this study may be the basis of an opportunity to introduce and conceptualize PC for the South Asian population in unique ways. In this regard, further research examining PC and end-of-life preferences for South Asian populations is required.

## Supplementary information

**Additional file 1.** Survey and semi-structured interview guide.

## Data Availability

The datasets used and/or analyzed during the current study are available from the corresponding author on reasonable request.

## Abbreviation

PC: Palliative Care
