# Supplementary material for: Perceptions of palliative care in a South Asian community: findings from an observational study
Source: BMC Palliat Care. 2020 Sep 14;19:141. doi: 10.1186/s12904-020-00646-6 (PMC7491098; doi:10.1186/s12904-020-00646-6)
Supplement: Supplementary file 1 — Additional file 1. Survey and semi-structured interview guide. [file 12904_2020_646_MOESM1_ESM.docx]

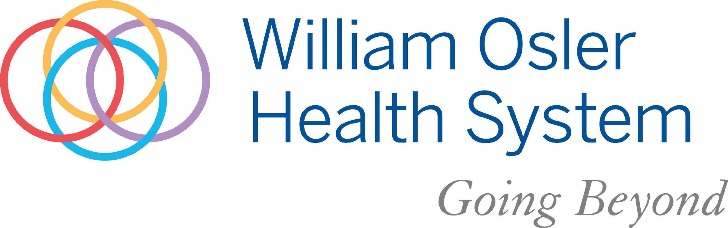


**Perceptions of Palliative Care in the South Asian Community**

**APPENDIX 1**

**SURVEY AND SEMI-STRUCTURED INTERVIEW GUIDE**

**Survey Questions**

1. **What is your preferred language of communication? (select one)**

English

Punjabi

Urdu

Hindi

Gujarati

Other (Please specify)_________________________________________________________

1. **What is your gender?**

- Male
- Female
- Other (please describe)__________________________________________________

1. **What is your age?**

- 18-29 years old
- 30-49 years old
- 50-64 years old
- 65-79 years old
- 80 years and over

1. **Which religious community do you consider yourself part of?**

- Hindu
- Muslim
- Christian
- Sikh
- Buddhist
- Jain
- None
- Other (please specify) ___________________________________________________

1. **What is your country of origin?**

- India
- Pakistan
- Bangladesh
- Sri Lanka
- Nepal
- Bhutan
- Other (please specify) ___________________________________________________

1. **If you are an immigrant to Canada, how many years have you lived in this country?**

- 0-5 years
- 6-10 years
- 11-15 years
- 16-20 years
- 20+ years

1. **What is the highest level of education you have completed?**

- Grade school
- Some high school
- High school graduate
- Some college / university
- Trade/technical/vocational training
- College / university graduate
- Some postgraduate work
- Post graduate degree
- No education

1. **What has your main occupation been in Canada? (please specify)**

**________________________________________________________________________**

1. **Have you ever heard of Palliative Care?**

- Yes
- No
- Unsure

**If Yes, then please identify level of knowledge:**

- Slightly knowledgeable
- Moderately knowledge
- Very knowledgeable

1. **There are many perceptions as to what palliative care means, which of the following matches your understanding of the term?**

- Stopping medical treatment only
- Comfort measures only
- Total care of a patient with a life limiting illness
- Symptom management of a life limiting illness
- Medical assisted aid in dying
- Other (please describe)__________________________________________________

1. **If you have heard of Palliative Care before receiving this survey, where did your information come from?**

- Not applicable
- Family Physician / Primary care provider discussed it
- Close friend or relative’s experience with Palliative Care
- Know of someone involved or received Palliative Care
- Conversation with friend / family / acquaintance
- Employment in health care
- Media (Radio, Television, Newspaper, Magazine, Website, Social media)
- Other (please describe)__________________________________________________

1. **What are, if any, the benefits you associate with palliative care? (check all that apply)**

- Better quality of life
- Less suffering
- Longer life span
- More resources provided for patient and family
- Hastened death
- Other (please describe)__________________________________________________

1. **What are, if any, the risks you associate with palliative care? (check all that apply)**

- Worsened quality of life
- More suffering
- Shorter life span
- Stopping treatment
- Giving up hope
- Other (please describe)__________________________________________________

1. **Where should a patient receive palliative care? (check all that apply)**

- Home
- Hospice
- Hospital
- Nursing home / Long-Term Care residence
- Other (please describe)_________________________________________________

1. **Who should provide palliative care? (check all that apply)**

- Nurse
- Doctor
- Family/partners/relatives
- Homecare providers/caregivers/ personal support worker
- Other (please specify)___________________________________________________

1. **Who should have the most say for palliative care decisions?**

- Family member
- Family Doctor
- Patients
- Specialist
- Caregiver/ Personal support worker/ homecare provider
- Other (please specify)___________________________________________________

1. **When would you want information about Palliative Care?**

- I would not want information at any point
- I would like more information to be generally available
- I would only want information to be provided if diagnosed with a life threatening illness
- I would only want information if a life threatening illness became a terminal diagnosis

1. **Do you think in your community you discuss death and dying:**

- Not enough
- About the right amount
- Too much

1. **Is Palliative Care consistent with your cultural values and/or faith/spiritual beliefs?**

- Yes
- No
- Unsure

1. **How important is religion or spiritual belief to you in Palliative Care?**

- Not Important
- Important
- Unsure

**Interview Guide**

***Interviewer introduction:***

Thank you for responding to our survey. I would now like to ask you a few more questions about yourself and what you think about palliative care. Your responses to these questions are being collected alongside your survey responses as data as part of the Perceptions in Palliative Care in the South Asian Community project.

There are 8 main questions that I will ask you. There is no right or wrong answer. It is your perspective that we would like to learn about. You can choose not to answer a question. If this is the case, please let me know and we will move on to the next one.

Do you have any questions?

***Elaboration on background knowledge of palliative care & definitional clarification:***

- Can you please describe to me what you know about palliative care?
- Where and when did you learn about palliative care?
- Palliative care is a perspective of care where we value quality of life and symptom control near end of life over length of life. For example, we use opioid medicines for shortness of breath although it may not help the underlying cause. Is this a type of care you would every see yourself receiving?
  - - If yes: Would you please tell me what you like about this approach?
    - If no: Would you please tell me what you do not like about this approach?

***Probing perceptions and ideals of care:***

Interviewer: I now have some questions to help us learn more about your perspectives on care, and about the kinds of supports you have.

- A: Who is caring for you right now? Who would you like to be caring for you at this time?
- B: Who would you expect to be caring for you near to the end of life?
  - From the answer they provide, probe for detail: Would you please tell me why this is?...
- Are you currently living at home?
  - Probe for detail RE housing situation, family arrangement: Are you living with family?...
- A and B: In the future, where would you prefer to spend your last days? I.e. home, hospital…
  - Probe for detail: Would you please tell me why this is?...
- A and B: In an ideal world, when would you first discuss end-of-life care options with your doctor?
- A and B: Would you please describe what a “good” death and end-of-life means to you?
